# Plasma IP-10 Concentrations Correlate Positively with Viraemia and Inversely with CD4 Counts in Untreated HIV Infection

**DOI:** 10.2174/1874613601711010024

**Published:** 2017-04-26

**Authors:** Kudakwashe Mhandire, Tommy Mlambo, Lynn Sodai Zijenah, Kerina Duri, Kudzaishe Mateveke, Mqondisi Tshabalala, Doreen Zvipo Mhandire, Cuthbert Musarurwa, Petronella Taonga Wekare, Lovemore Ronald Mazengera, Hilda Tendisa Matarira, Babill Stray-Pedersen

**Affiliations:** 1 Department of Chemical Pathology, University of Zimbabwe, Harare, Zimbabwe; 2 Department of Immunology, University of Zimbabwe, Harare, Zimbabwe; 3 Medical Laboratory Sciences, University of Zimbabwe, College of Health Sciences, Harare, Zimbabwe; 4 Letten Foundation Research House, Harare, Zimbabwe; 5 Institute of Clinical Medicine, University of Oslo and Womens’ Clinic, Rikshospitalet, University Hospital, Oslo, Norway; 6 Research Support Centre, University of Zimbabwe, College of Health Sciences, Harare, Zimbabwe

**Keywords:** Immune activation, sCD14, IP-10, CD4 count, Viral load, Zimbabwean

## Abstract

**Background:**

Chronic immune activation is a feature of HIV infection associated with accelerated HIV disease progression. There is conflicting data on the association of biomarkers of immune activation with traditional markers of HIV disease progression; CD4 counts and viral load (VL).

**Objective:**

The study aimed to determine the association of biomarkers of immune activation; interferon (IFN)-γ-induced protein 10 (IP-10) and soluble cluster of differentiation 14 (sCD14) in chronic HIV infection with traditional markers of HIV disease progression.

**Methods:**

We collected demographic data, enumerated CD4 counts and quantified VL in 183 antiretroviral therapy (ART)-naive adults with chronic HIV infection. Plasma concentrations of IP-10 and sCD14 were quantified in the ART-naive adults with chronic HIV infection and 75 HIV-uninfected controls.

**Results:**

IP-10 concentrations were significantly higher in the HIV-infected group (median; 257.40pg/ml, IQR; 174.08-376.32) than in the HIV-uninfected (median; 86.19pg/ml, IQR; 67.70-116.39) (P<0.001). Similarly, sCD14 concentrations were significantly higher in the HIV-infected (median; 1.45µg/ml, IQR; 1.02-2.16) group than in the controls (median; 0.89µ/ml, IQR; 0.74-1.18) (P<0.001). High log_10_ IP-10 concentrations were positively correlated with high log_10_ viral loads (Spearman’s correlation coefficient [R]=0.21, P=0.003) and inversely correlated with low CD4 counts (R= -0.19, P=0.011). In contrast, log_10_ sCD14 was not significantly associated with either log_10_ viral loads (R=0.03, P=0.707) nor CD4 count (R=-0.04, P=0.568).

**Conclusion:**

We conclude that plasma sCD14 and IP-10 were elevated in the HIV-infected patients compared to HIV-uninfected individuals possibly due to on-going immune activation. In addition, plasma high concentrations of IP-10 but not sCD14 concentrations are associated with high VL and low CD4 count.

## INTRODUCTION

Chronic immune activation is a feature of HIV infection that is associated with accelerated HIV disease progression [1]. Chronic immune activation is the persistent stimulation of the immune system characterized by elevation of markers of cell activation, cytokines, acute phase proteins and pathological changes in composition of immune cells [2]. HIV replication, co-infections, depletion of gut mucosal integrity and consequent microbial translocation are among the main mechanisms of immune activation in HIV infection [3]. In non-human primates that do not progress to AIDS despite high simian immunodeficiency virus (SIV) load, immune activation that follows infection is transient and often successfully reduced to pre-infection levels without intervention [2]. In contrast, immune activation tends to be chronic among non-human primates SIV and human HIV disease progressors, suggesting that immune activation may be the major vehicle behind disease progression. However, the dynamics of immune activation in HIV infection are not yet fully understood.

ART down-regulates immune activation but does not eradicate it [4]. Thus, immune activation is reported to persist despite successful viral suppression [5] leading to immune depletion and non-AIDS complications such as cardiovascular disease and kidney disease [6, 7]. Efforts to identify biomarkers that correlate with immunological, virological and clinical disease progression have been informative but contradictory [2, 8]. Among the plasma biomarkers documented to date, IP-10, a chemokine secreted by leukocytes, monocytes, epithelia, stromal cells and keratinocytes following induction by IFN- γ [9], and sCD14, a cell receptor released from monocytes stimulated by lipopolysaccharides during microbial translocation [8], hold promise for use as markers of immune activation and possibly disease progression. However, results from studies comparing IP-10 and sCD14 levels in the HIV- infected individuals and uninfected individuals are contradictory [1, 7, 10-12]. Research findings on the association between IP-10 and sCD14 concentrations and VL and/or CD4 counts are also controversial [1, 10, 12-14]. Persistence of immune activation despite viral suppression increases the risk of immunological depletion and development of non-AIDS events, hence monitoring of immune activation may be important in HIV management.

In this study, we described differences in the markers of immune activation, IP-10 and sCD14 between ART-naïve HIV-infected and HIV-uninfected adult Zimbabweans. In addition, we correlate IP-10 and sCD14 concentrations with VL and CD4 count to identify their role in HIV disease progression. ART-naive HIV-infected individuals present us with an opportunity to determine the interaction between immune activation and virological/immunological status. We found both IP-10 and sCD14 elevated in the HIV-infected group compared to HIV-uninfected controls. However, only high concentrations of IP-10 were positively correlated with high VL and inversely correlated with low CD4 count.

## METHODS

### Study Participants

HIV-infected, ART naïve Black Zimbabweans aged ≥ 18 years with CD4 count >350cells/µL were enrolled into the Immunological and Virological Investigations of HIV-infected individuals with CD4 counts >350 cells/µL (IVIHIV) Study at the Parirenyatwa Groups of Hospitals Opportunistic Infections Clinic in Harare, Zimbabwe. Written informed consent was obtained from each participant. The study was conducted from 2010 to 2013. During this period, ART was provided only to patients with CD4 counts <350cells/µL under the Zimbabwe National ART Program. At enrollment, a questionnaire was administered to collect demographic data and medical history, whole blood was collected in ethylene diaminetetraacetic acid (EDTA) anti–coagulant for CD4 counts and plasma isolation within six hours of collection. The plasma was immediately stored at -80^0^C until further use. Plasma HIV RNA load (VL) was quantified using the archived plasma collected at enrolment. The median time since the diagnosis of HIV infection in the cohort was 6.3 years, hence the patients were considered as chronically HIV-infected.

In this cross sectional study, we quantified IP-10 and sCD14 in the 183 HIV-infected individuals using archived plasma collected at enrolment. We then determined the association of the concentrations of these two biomarkers with viral load and CD4 counts. We also collected demographic information and EDTA whole blood from 75 unmatched HIV-uninfected Black Zimbabwean blood donors aged ≥ 18 years at the National Blood Transfusion Services (NBTS) of Zimbabwe in Harare who provided written informed consent to participate in the study. The study was approved by the Medical Research Council of Zimbabwe (MRCZ/A/2016).

### Measurement of Plasma IP-10 and sCD14 Concentrations

IP10 and sCD14 concentrations were measured in archived plasma from HIV-infected cases and HIV-uninfected controls using Quantikine^®^ enzyme linked immune-sorbent assays (R&D Systems, Minneapolis, USA) following the manufacturer’s instructions. Each assay was run in duplicate. The lower detection limits of the assays were 0.125µg/mL and 1.67pg/mL for sCD14 and IP-10, respectively.

### Measurement of CD4 Counts and Plasma Viral Load

CD4+ T lymphocytes were enumerated in EDTA anti coagulated whole blood within 6 hours of blood collection using the Partec platform (Sysmex, Germany) the following manufacturer’s instructions. HIV RNA copies were estimated in archived plasma using the Cavidi Exavir^TM^ viral load version 3 assay (Cavidi AB, Uppsala Science Park, Sweden) following the manufacturer’s instruction. The VL assay has a lower detection limit of 200 copies/ml.

### Statistical Analyses

Statistical analyses were done using Stata version 13.0 (StataCorp, Texas, USA) and GraphPad Prism 7 (GraphPad Software Inc, Carlifornia, USA). Demographic and clinical characteristics were compared between HIV-infected and HIV-uninfected groups using the Student’s T-test for parametric variables, Mann-Whitney sum rank for non-parametric variables, and Chi-squared test or Fisher’s exact tests for categorical data. A value of P<0.05 was considered statistically significant. P-values were adjusted for multivariate comparisons using Bonferroni correction method. For correlation analysis, a p-value <0.025 was considered significant. Spearman’s correlation coefficient (R) was used to infer association between biomarkers and traditional markers of HIV disease progression (VL and CD4 count). A multivariate linear regression analysis was used to predict the effect of age, gender, weight and co-morbidities on IP-10 and sCD14 concentrations in the HIV-infected group and that of age, gender and weight in the HIV-uninfected group.

## RESULTS

### Demographic and Clinical Characteristics of the Participants

The demographic characteristics of the participants are shown in Table (**1**). The mean weight of the HIV-infected participants was significantly lower than that of the HIV-uninfected participants. Females were overrepresented in the HIV-infected group than in the uninfected group. Mean age was not significantly different between the HIV-infected and uninfected participants. Twenty-six (14%) participants had VL ≤200cpm; 55 (30%) had VL >200cpm but ≤1000cpm, 64 (35%) >1000cpm but ≤10000cpm whilst the remaining 38 (21%) had VL >10000cpm. The median CD4 count was 435 cells/µl, IQR: 388-556. One hundred and twenty-four (68%) participants had CD4 counts <500 cells/µl (median: 407 cells/µl, IQR: 309-496) while 59 (32%) had ≥500 cells/µl (median: 613 cells/µl, IQR: 503-923). Fifty-one (28%) of the HIV-infected individuals had at least one self reported co-morbidity, of whom 15 (30%), had two or more co-morbidities.

### Plasma IP-10 and sCD14 Concentrations in HIV-Infected and HIV-uninfected Groups

We compared IP-10 and sCD14 plasma concentrations between HIV-infected cases and HIV-uninfected controls to determine association between the concentrations of these biomarkers with HIV status Fig. (**1**). IP-10 plasma concentrations were significantly higher in the HIV-infected group (median; 257.40pg/ml, IQR; 174.08-376.32) than in the HIV-uninfected (median; 86.19pg/ml, IQR; 67.70-116.39) (P<0.001). Similarly, sCD14 plasma concentrations were significantly higher in the HIV-infected (median; 1.45µg/ml, IQR; 1.02-2.16) group than the HIV-uninfected (median 0.89µg/ml, IQR; 0.74-1.18) (P<0.001). There was no significant correlation between IP-10 and sCD14 concentrations in either the HIV-infected (R = 0.09, P=0.248) or the HIV-uninfected groups (R= 0.05, P=0.662). We used a linear regression analysis to account the possible effect of age, gender and weight between the HIV-infected and uninfected groups on IP-10 and sCD14 concentrations. Age, gender and weight were not significantly associated with either IP-10 or sCD14 plasma concentrations in either the HIV-infected or HIV-uninfected groups (Supplementary Table **1**). Also, having an HIV co-morbidity was not associated with either IP-10 or sCD14 concentrations.

### Correlation of IP-10 and sCD14 with VL and CD4 Counts.

We then determined the correlation between immune activation biomarkers (IP-10 and sCD14) and traditional markers of HIV disease progression (VL and CD4 counts). As shown in Fig. (**2**), high log_10_ IP-10 concentrations were positively correlated with high log_10_ viral loads (R=0.21, P=0.003) and inversely correlated with low CD4 counts (R= -0.19, P=0.011). However, log_10_ sCD14 was not associated with either log_10_ viral load (R=0.03, P=0.707) or CD4 count (R= -0.04, P=0.568).

## DISCUSSION

Immune activation is an important determinant of HIV disease course. Here, we characterized biomarkers of immune activation (IP-10 and sCD14) in HIV-infected patients and HIV-uninfected individuals in Zimbabwe. The plasma IP-10 and sCD14 concentrations were elevated in the HIV-infected group compared to the HIV-uninfected group. Among the HIV-infected individuals, high plasma IP-10 concentrations were positively correlated with high VL and inversely correlated with low CD4 count. Surprisingly, there was no significant correlation of plasma sCD14 with either plasma VL or CD4 count. Our findings support the existence of chronic immune activation in chronic HIV-infection as previously described [2, 15] and the positive correlation of high IP-10 concentrations with high VL and inverse correlation with low CD4 count. To our knowledge, this is the first study to determine IP-10 and sCD14 plasma concentrations as biomarkers of immune activation and their association with traditional markers of HIV disease progression among Zimbabweans infected predominantly with HIV-1 subtype C.

Our findings are consistent with earlier reports of significant elevation of plasma IP-10 [1, 10, 14, 16] and sCD14 [7, 16, 17] in HIV-infected adults compared to HIV-uninfected controls. IP-10 is among the earliest cytokines to become elevated following infection with HIV [18]. Plasmacytoid dendritic cells (pDC) recruited to a site of infection produce IFNs that induce IP-10 production [14] by a range of cells that includes leukocytes, monocytes and neutrophils [13]. While this is an active mechanism for IP-10 production in acute HIV infection stage, our study participants had chronic HIV infection. In chronic HIV infection, prolonged production of IFN-γ in lymphoid organs is responsible for sustained IP-10 elevation [19]. Despite marked reduction in plasma IP-10 from acute HIV infection stage concentrations, chronic HIV infection concentrations remain higher than pre-infection concentrations even in patients commenced on ART [20]. IP-10 is a ligand of the CXCR3 receptor which is not known to function as a co-receptor for HIV [21], however, CXCR3+ CD4+ T lymphocytes show high HIV infection rate [22]. Furthermore, IP-10 increases susceptibility of nascent naïve CD4+ T lymphocytes to HIV infection [23], hence IP-10 enhances the establishment of HIV reservoirs [12].

Elevated concentrations of sCD14 in HIV-infected compared to HIV-uninfected individuals are largely attributed to monocyte activation by bacterial lipopolysaccharides (LPS) leaking into circulation through compromised gut mucosa, a process known as microbial translocation [8]. CD14 molecules are expressed on monocytes where they act as co-receptors for LPS and other microbial components [24]. When LPS bind CD14 receptors, monocytes are activated to release CD14 in a soluble form, sCD14 [24] hence the rise in plasma sCD14. Higher concentrations of sCD14 in HIV-infected individuals compared to the HIV-uninfected have also been reported in other studies [1, 25, 26]. Microbial translocation as determined by elevated sCD14 has been linked with increased mortality among HIV-infected individuals [25] and individuals on haemodialysis [27]. Importantly, sCD14 concentrations are also elevated in hepatitis C virus infection [28], tuberculosis [29], inflammatory disorders such as rheumatoid arthritis and systemic lupus erythematosus [30] and in HIV associated accelerated aging [31]. The diversity of conditions in which sCD14 levels are reported elevated perhaps limits the specificity of the biomarker to any of them thereby limiting its utility as a diagnostic or prognostic marker. Interestingly, although, high levels of sCD14 were mostly associated with poor prognosis in these conditions. In the current study, we did not observe any association with traditional markers of HIV disease progression.

In sharp contrast, we found IP-10 concentrations positively and negatively correlated to VL and CD4 counts, respectively. These findings corroborate reports from earlier studies [1, 8-11]. Whilst our study focused on IP-10 and sCD14 because of their link to HIV status and markers of disease progression described in literature, some studies have profiled between 15 and 28 proteins and singled out elevated IP-10 concentrations to positively correlate with high VL and inversely correlate with low CD4 counts [1, 10, 12-14]. Determination of IP-10 concentrations may have a prognostic value in early HIV infection as elevated IP-10 concentrations have been reported to predict disease progression and up to 30% variation in CD4 count set point [10, 19]. However, it remains unclear whether measurement of plasma IP-10 may be useful in HIV patient management during the acute or chronic stages of infection. In chronic HIV infection, immune activation leads to poor prognosis and eventual death [15, 32] hence we speculate that biomarkers such as IP-10 whose elevation may favor viral replication may guide anti-immune activation treatment in future.

IP-10 stimulates viral replication in infected cells [21] consequently increasing viraemia. In the vaginal mucosa, an important site in HIV pathogenesis, IP-10 is associated with increased HIV shedding [33] that facilitates infection of new targets. Persistent elevation of IP-10 has been linked to immunological treatment failure among patients on cART despite suppressed viraemia [20]. Immune activation therefore threatens the gains of ART hence treatment options aimed at reducing immune activation have been explored, but with mixed results [15]. Importantly, though statistically significant, the correlations between IP-10 and VL and CD4 counts in our study were fairly weak hence our findings need to be interpreted with care. Our cross sectional approach may have precluded investigations on the influence of IP-10 and sCD14 on HIV disease progression. A longitudinal study involving multiple time point measurements of IP-10 and sCD14 against VL and CD4 counts from acute infection stage to chronic stage is therefore recommended. However, accurate timing of HIV infection remains a challenge because patients tend to seek treatment when they have reached the chronic stage. Furthermore, differences between HIV-infected patients and voluntary blood donors may have introduced some selection bias and other undetectable confounding to our findings.

## CONCLUSION

IP-10 and sCD14 concentrations were elevated in the HIV-infected patients compared to HIV-uninfected individuals possibly due to on-going immune activation. In addition, plasma high concentrations of IP-10 but not sCD14 concentrations were associated with high VL and low CD4 count. Thus, IP-10 secretion may be associated with HIV pathogenesis and immune depletion. Prospective studies with larger sample sizes are required to establish causation.

## Figures and Tables

**Fig. (1):**
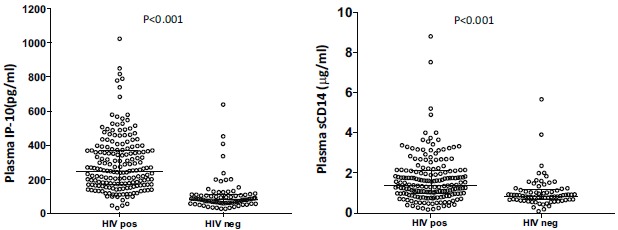
Comparison of plasma IP-10 and sCD14 levels between HIV-infected and HIV-uninfected groups. Mann-Whitney rank sum test was used to compare difference between medians of the two groups. Both IP-10 and sCD14 were significantly higher in the HIV-infected group than the HIV-uninfected group.

**Fig. (2):**
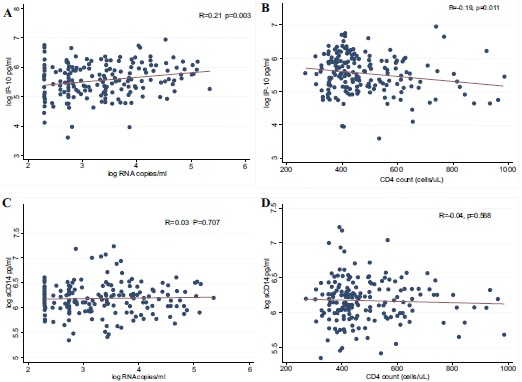
Correlation of IP-10 and sCD14 with viral loads and CD4 counts. Spearman’s correlation coefficient was used to determine strength of association between biomarkers (log10 IP-10 and log10 sCD14) and markers of disease progression (log10 viral loads and CD4 counts). Log10 IP-10 levels positively correlate with log10 viral load (A) and negatively with CD4 count (B). CD14 levels were not significantly associated with neither log10 viral load (C) nor CD4 count (D).

**Table 1:** Demographic and clinical characteristic of participants.

Characteristic	HIV-infected (n=183)	HIV-uninfected (n=75)	p-value
Female n (%)	136 (0.74)	29 (0.39)	<0.001*
Mean age in years ± SD	34.7±9.1	36.9±11.8	0.08^a^
Mean weight in kg ± SD	66.8±0.98	75.6±9.8	<0.001^a^
Median CD4 count in cells/µl (IQR)	435 (388-556)	NA	NA
Median plasma viral load in copies/mL (IQR)	1444 (519-7858)	NA	NA
≥ 1 current co-morbidities	51(0.28)	NA	NA

SD: standard deviation; IQR: interquartile range; NA: non-applicable; *Chi-squared test; ^a^Student’s T-test.

**Supplementary Table 1:** Summary of simple linear regression analyses of demographic variables predicting plasma IP-10 and sCD14 concentrations in the HIV-infected and HIV-uninfected groups.

	**Coefficient**	**Standard Error**	**P-value**
**HIV-infected**			
*For Log IP-10*			
Gender	0.153	0.101	0.131
Age	0.005	0.005	0.250
Weight	0.002	0.003	0.554
Co-morbidity	-0.078	0.098	0.423
*For log sCD14*			
Gender	0.074	0.056	0.196
Age	0.002	0.002	0.575
Weight	0.002	0.003	0.947
Co-morbidity	-0.003	0.055	0.963
**HIV-uninfected**			
*For Log IP-10*			
Gender	-0.098	0.148	0.508
Age	-0.007	0.008	0.318
Weight	0.005	0.009	0.594
*For log sCD14*			
Gender	-0.074	0.057	0.196
Age	-0.001	0.002	0.575
Weight	0.001	0.003	0.947
